# Leveraging multisectoral approach to understand the determinants of childhood stunting in Rwanda: a systematic review and meta-analysis

**DOI:** 10.1186/s13643-023-02438-4

**Published:** 2024-01-05

**Authors:** Chester Kalinda, Maria Albin Qambayot, Sage Marie C. Ishimwe, Denis Regnier, Darius Bazimya, Theogene Uwizeyimana, Samson Desie, Christiane Rudert, Alemayehu Gebremariam, Elizabeth Brennan, Silver Karumba, Rex Wong, Abebe Bekele

**Affiliations:** 1 Bill and Joyce Cummings Institute of Global Health, University of Global Health Equity, Kigali Heights, Plot 772 KG 7 Ave, PO Box 6955, Kigali, Rwanda; 2 Centre for One Health, University of Global Health Equity, Kigali Heights, Plot 772 KG 7 Ave, PO Box 6955, Kigali, Rwanda; 3 Institute of Global Health Equity Research (IGHER), University of Global Health Equity, Kigali Heights, Plot 772 KG 7 Ave, PO Box 6955, Kigali, Rwanda; 4 School of Medicine, University of Global Health Equity, Kigali Heights, Plot 772 KG 7 Ave, PO Box 6955, Kigali, Rwanda; 5 UNICEF Kigali Office, P.O. Box 381, Kigali, Rwanda; 6 UNICEF ESARO, Gigiri, PO Box 44145-00100, Nairobi, Kenya; 7 Health Office, US Agency For International Development (USAID), Rwanda Office, KG 7 Avenue, Kigali, Rwanda; 8 Catholic Relief Services, Rwanda Country Program, Chadel Building, P.O. Box 65, Kigali, Rwanda

**Keywords:** Rwanda, Child health, Stunting, Demographic and Health Surveys, Meta-analysis

## Abstract

**Background:**

Addressing childhood stunting is a priority and an important step in the attainment of Global Nutrition Targets for 2025 and Sustainable Development Goals (SDGs). In Rwanda, the prevalence of child stunting remains high despite concerted efforts to reduce it.

**Methods:**

Utilizing the United Nations International Children’s Emergency Fund (UNICEF) framework on maternal and child nutrition, this study systematically evaluated the determinants of child stunting in Rwanda and identified available gaps. Twenty-five peer-reviewed papers and five Demographic and Health Surveys (DHS) reports were included in the final selection of our review, which allowed us to identify determinants such as governance and norms including wealth index, marital status, and maternal education, while underlying determinants were maternal health and nutrition factors, early initiation of breastfeeding, water treatment and sanitation, and immediate factors included infections.

**Results:**

A total of 75% of the overall inequality in stunting was due to the difference in the social determinants of stunting between poor and nonpoor households. Maternal education (17%) and intergenerational transfer (31%) accounted for most of the inequalities in stunting, and an increase in gross domestic product per capita contributed to a reduction in its prevalence. There is a paucity of information on the impact of sociocultural norms, early life exposures, maternal health and nutrition, and Rwandan topography.

**Conclusion:**

The findings of this study suggest that improving women’s status, particularly maternal education and health; access to improved water, sanitation, and hygiene-related factors; and the socioeconomic status of communities, especially those in rural areas, will lay a sound foundation for reducing stunting among under-5 children.

**Supplementary Information:**

The online version contains supplementary material available at 10.1186/s13643-023-02438-4.

## Introduction

Undernutrition continues to be a global health challenge that affects millions of people, especially women, children, and other marginalized populations. According to the FAO, IFAD, UNICEF, WFP, and WHO report of 2019, over 820 million people suffer from hunger [1], of which over 250 million are from Africa, where the rate of rise in the number of undernourished people is higher than any other part of the world [2]. In 2015, United Nations member countries set out an ambitious agenda in the Sustainable Development Goals (SDGs) of ending poverty and improving the lives and prospects of everyone by 2030 [3, 4]. SDG 2 focuses on “zero hunger” achievable by “eradicating hunger” and “enabling access to safe, nutritious, and sufficient food by all people year-round” (SDG Target 2.1) [5], suggesting the potential impact of effective nutrition interventions.

Improving nutrition is at the core of global development and is central to achieving the SDGs. SDG 2 recognizes the imperative for better nutrition and aims to “end hunger, achieve food security and nutrition, and promote sustainable agriculture” [2, 5, 6]. However, improving nutrition goes beyond achieving only SDG 2. It is linked to all SDGs and thus plays an essential transformational role in driving them [7]. This is a two-way interaction in which the battle against undernutrition will have wide-reaching consequences for improving human capital development and ultimately contribute to the achievement of the SDGs. Similarly, achieving nutritional goals will depend on progress across many other SDGs, including those aimed at poverty reduction, clean water and sanitation, education, and gender equality [8, 9].

Studies conducted in sub-Saharan countries (see Akombi et al. [10], Woldeamanuel and Tesfaye [11], Dapi Nzefa et al. [12], Tesema et al. [13]) indicate that many children experience undernutrition (stunting, wasting, and underweight), overnutrition (overweight/obesity), and micronutrient deficiencies leading to a possible triple burden of malnutrition. In the COVID-19 and emerging and re-emerging infections era, this burden has increased the strain on healthcare systems and expenditure, increasing preventable morbidities and mortality [14–18]. The goal of reducing the prevalence of malnutrition and its associated morbidities has led several governments and partner organizations in sub-Saharan Africa (SSA) to formulate improved strategies and interventions to effectively address this problem [19–21]. Although these strategies and interventions have been effective, several challenges remain, ranging from the lack of reliable data on the prevalence and determinants of malnutrition to barriers to sustainable program implementation.

In the aftermath of the genocide against the Tutsi and during the post-genocide reconstruction of the country, Rwanda developed an innovative health system that improves access to quality primary healthcare through its community-based health insurance plan [22]. In 2014, Rwanda implemented a National Food and Nutrition Policy to address the persistently high prevalence of child stunting [21]. Despite the spectacular gains in health outcomes, some studies (see Habyarimana et al. [23], Nshimyiryo et al. [24], Binagwaho et al. [22]) suggested that undernutrition especially stunting remains a serious health burden, affecting one-third of children below the age of 5 years. To achieve Rwanda’s Vision 2050 targets of reducing stunting to 5.5% by 2035 and to 3% by 2050 and attaining the SDGs, addressing persistent stunting by designing new, innovative, evidence-based, and sustainable strategies is key. In this study, we reviewed the available literature and extracted data from studies conducted in Rwanda to identify the determinants of persistent stunting.

## Methods

**Fig. 1:**
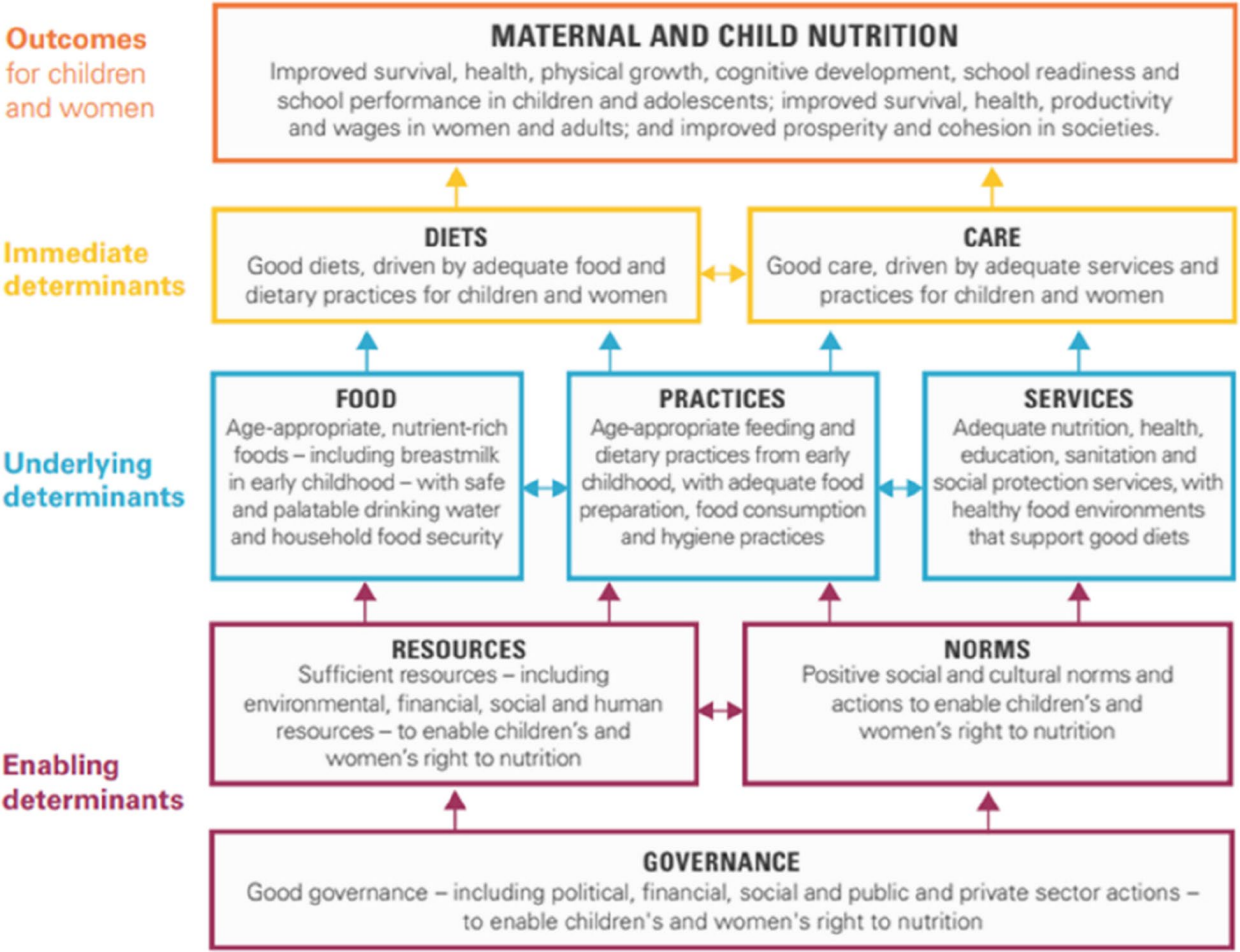
UNICEF conceptual framework on the determinants of maternal and child nutrition

A systematic review and meta-analysis of peer-reviewed and gray literature published between 1 January 2000 and 31 January 2023, as well as secondary data analysis of the last five consecutive DHS published in the same period, were performed using the Preferred Reporting Items for Systematic Reviews and Meta-analyses (PRISMA) [25]. The UNICEF conceptual framework on maternal and child nutrition (Fig. 1) was used to extract data on the determinants from the selected papers based on the Rwandan context, considering that the causes and contextual factors identified in the framework were based on global data.

### Eligibility criteria

This review included prospective and retrospective cohort and cross-sectional studies that focused on malnutrition among children under the age of 5 years, with a sample size of 10 or more. Studies that had the clinical definition of malnutrition as defined by the DHS [26] and WHO growth standards were included [27]. Studies with no details of the data sources or study population ages were excluded from the analysis.

Rwanda has had seven cycles of DHS, the latest being the 2020 cycle. In our analysis, we excluded DHS-based articles published during the 1992 and 1996 cycles because of the political instability of the country. Rwanda experienced civil unrest in 1990, followed by genocide against the Tutsi in 1994, leading to the death of more than one million people and a devastated national economy which had already been precarious [28]. Efforts to rebuild the country started from 1999 onwards, and broad social and health reforms were made, including strengthening all health system components [29–32]. Thus, we were confident that including articles published between 1 January 2000 and 31 January 2023 would provide sufficient information and trends to demonstrate changes in the determinants of malnutrition among children under 5 years of age in Rwanda.

### Data sources and search strategies

The primary outcome of this review was malnutrition and the factors that had been assumed to influence malnutrition. Our study observed that most studies used secondary DHS data; thus, we adopted the UNICEF conceptual framework on maternal and child nutrition [33] to guide the extraction of variables of interest. UNICEF’s conceptual framework approach to understanding malnutrition is comprehensive and resonates with the intricacies of the Rwandan scenario. This framework considers the multidimensional nature of malnutrition, since stunting is more than just insufficient food intake but a complex interplay of various factors, such as health services, hygiene practices, and socio-economic conditions (Supplementary file 1). We developed a search strategy using key concepts from DHS reports and the UNICEF conceptual framework for anthropometric assessment, malnutrition, and Rwanda. For each key concept, we designed suitable free-text words, and Medical Subject Headings (MeSH) were used in combination with Boolean operators, such as AND and OR. Our initial search led to the retrieval of 4511 articles, 4 webpages, and 6 project reports.

**Fig. 2:**
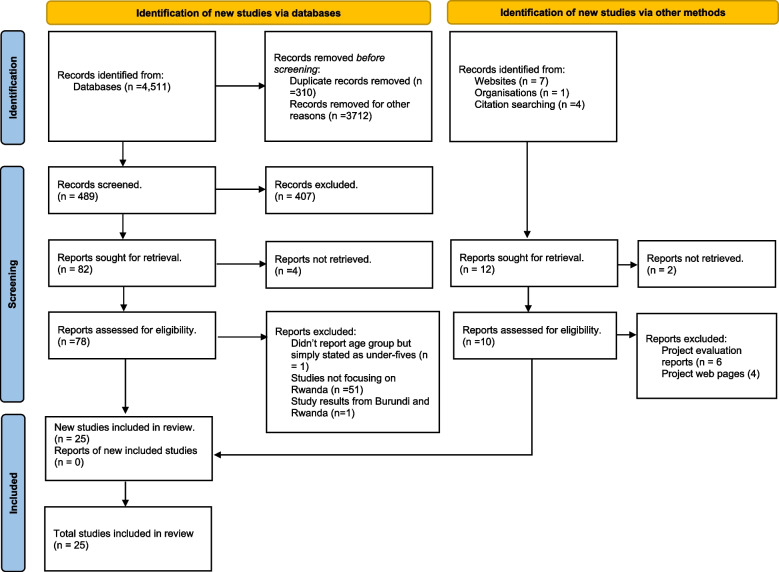
PRISMA flow diagram for studies which included searches of databases and other sources

Our initial search strategy involved pretesting the terms in PubMed by two independent reviewers (MAQ and SMCI), while disagreements arising from the two reviewers were resolved by a third reviewer (C. K.). This was followed by a search for relevant literature in different electronic databases, such as CINAHL (EBSCO), MEDLINE (via Ovid), and PubMed, performed between 07 December 2022 and 31 January 2023. We further searched Google Scholar to identify any gray literature related to malnutrition in Rwanda. By snowballing and scanning the reference lists of papers that had been identified as relevant, we also searched government websites to further identify documents relevant to the study. The search strategy and literature retrieval are presented in the PRISMA diagram in Fig. 2.

### Study selection

All studies identified from various electronic databases with potentially eligible data for inclusion were exported to EndNote version 20, and all duplicates were removed. A three-phase screening process was used. First, the titles and abstracts of the remaining citations in EndNote were screened by two independent reviewers, and all ineligible studies based on their titles and abstracts were excluded. Thereafter, a full-text review of the remaining articles was performed to ascertain their suitability for inclusion before data extraction.

### Data extraction process and quality assessment

Data extraction was based on the UNICEF conceptual framework for maternal and child nutrition [33]. The framework categorizes child nutrition as an outcome of three major components, with one layer influencing others. The conceptual framework identifies enabling factors, including governance, resources, and societal norms, as well as the underlying factors (food, practices, and services) that influence immediate determinants (diet and care) which ultimately affect child nutrition (Fig. 1). The quality of all studies included in the final selection was assessed using an adapted tool from the Joanna Briggs Institute checklist for analytical cross-sectional studies [34]. The quality of each study was assessed based on eight quality control items, and a score of 1 was assigned for each fulfilled item; otherwis (e, 0. Aggregate scores were used to generate a quality index for each study, and the quality was classified as low (0.0–0.4), moderate (0.5–0.7), or high (0.8–1.0) (Supplementary file 2).

Data extraction of the determinants of interest was based on an Excel-based standardized data abstraction form. Data extraction was carried out by two reviewers (M. Q. A. and S. M. C. I.), and any disagreements arising from the included variables or transformation of the observed strength of association between the abstracted variables and stunting to odds ratios were resolved by CK. The study further extracted and synthesized information from the World Bank relating to the gross domestic product per capita in Rwanda. Furthermore, we explored how certain factors can be transferred from one generation to another and how this intergenerational transfer of factors can influence stunting. To understand this, which often results from socioeconomic inequities, we adopted the definition of intergenerational transfer from Von Fintel and Richter [35].

Quantitative data were analyzed using MetaXL, an Excel-based meta-analysis tool. MetaXL software was chosen because it employs a more robust analytical approach, inverse variance heterogeneity (IVhet), in addition to the fixed effect (FE) and random effects (RE) models, while it also includes the Doi plot as a new method for the detection of publication bias [36, 37]. The IVhet maintains a correct coverage probability at a lower detected variance model regardless of heterogeneity [37]; thus, there is no underestimation of the statistical error, and it maintains modest estimates. The association between the determinants and stunting was reported as an odds ratio, their occampaying *p*-values, and 95% confidence interval (CI). The results are graphically presented using the using forest plots. The presence of heterogeneity and publication bias was assessed using Cochran’s Q statistic and I^2^ Doi plot, respectively [38], while publication bias was assessed using the Luis Furuya–Kanamori (LFK) index of the Doi plot [37]. The symmetry of Doi plots was determined using the LFK index, and the magnitude of the index was used to ascertain publication bias. An index value in the range of “ ± 1” was classified as “symmetrical” and thus the “absence of publication bias.” An index value of “ ± 2” was classified as “minor asymmetry” thus “low publication bias.” LFK index values outside the range of “ ± 2” were classified as “major asymmetry” thus “high publication bias” [37]. To explore heterogeneity and factors that could potentially influence the observed association, subgroup analysis was performed by stratifying our data according to child, maternal, household, water, sanitation and hygiene characteristics, and infection. We further performed a sensitivity analysis by excluding variables based on their level of significance and assessed the model significance based on forest plots.

## Results

The final review included 25 studies that used primary and secondary data (Supplementary file 3). Of these, enabling determinant factors, such as governance and norms, included the wealth index and marital status. Underlying determinants included maternal factors, such as maternal education, early initiation of breastfeeding, water treatment, and sanitation, while immediate factors included infections. Our results indicate that 75% of the overall inequality was due to the difference in the social determinants of stunting between poor and nonpoor households. Of these, intergenerational transfer accounted for 31%, education for 17%, inadequate care and lack of health services for 12.2%, household food insecurity for 10.2%, unhealthy household environment for 3.6%, and diseases for 0.6%. However, 25.5% of the inequalities in stunting remained unexplained.

**Fig. 3:**
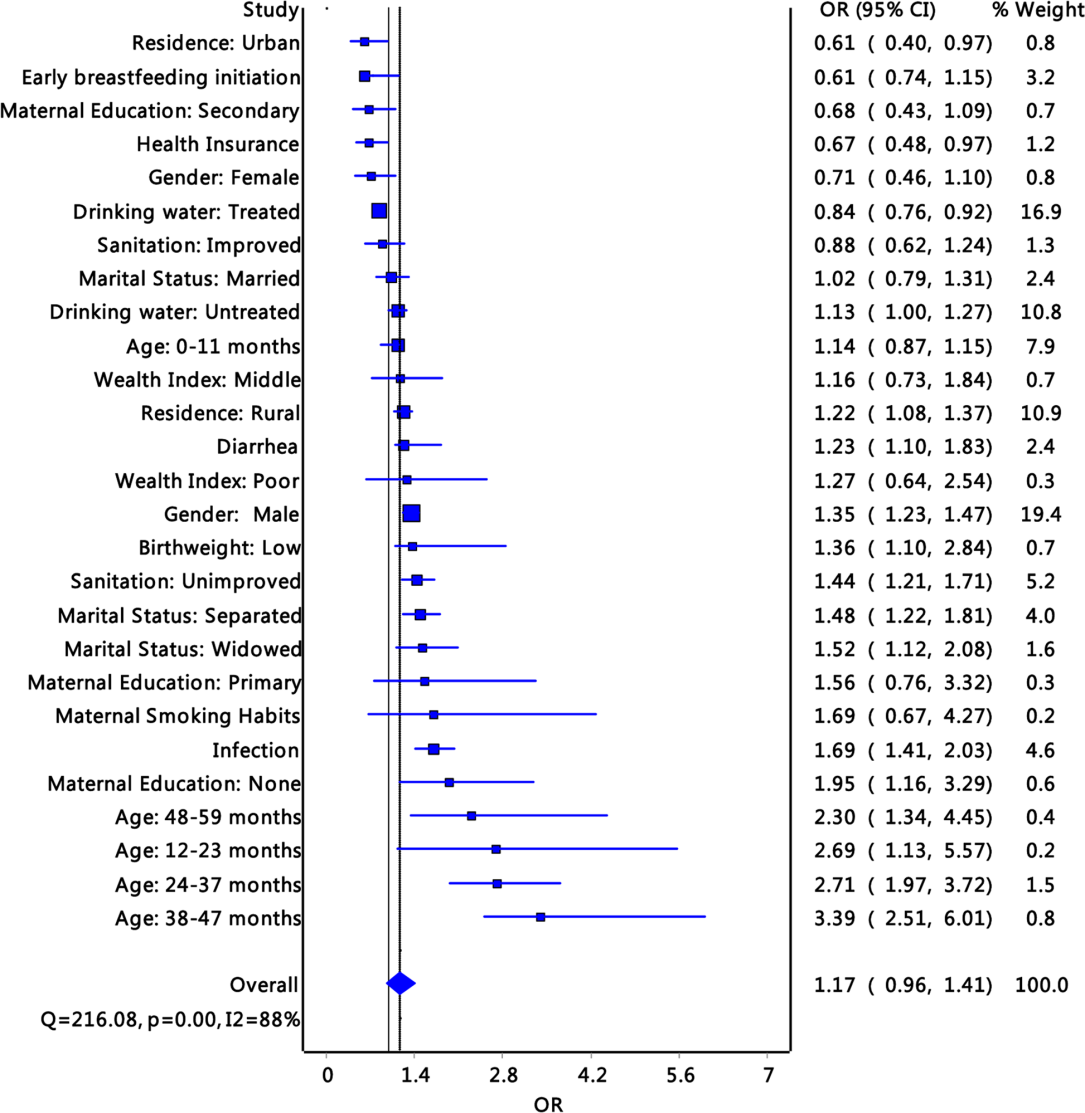
Forest plots of pooled determinants of stunting

Importantly, the influence of resources on residence such as rural residence (*OR*: 1.22; 1.08–1.37) increased the likelihood of stunting. Underlying causes such as unimproved sanitation (*OR*: 1.44, *p* < 0.01; 95% *CI*: 1.21–1.71) and infection among children (*OR*: 1.69; *p* < 0.01; 95% *CI*: 1.41–2.03) increased the likelihood of stunting. Other determinants of stunting included age, with stunting peaking at 38–47 months (*OR*: 3.39; *p* < 0.01; 95% *CI*: 2.51–6.01) before reducing among those aged 48–59 months (*OR*: 2.3; *p* < 0.023; 95% *CI*: 1.34–4.45) (Fig. 3). Attainment of higher education among mothers, early initiation of breastfeeding, having medical insurance, improved sanitation, and using treated drinking water were protective against stunting.

**Fig. 4:**
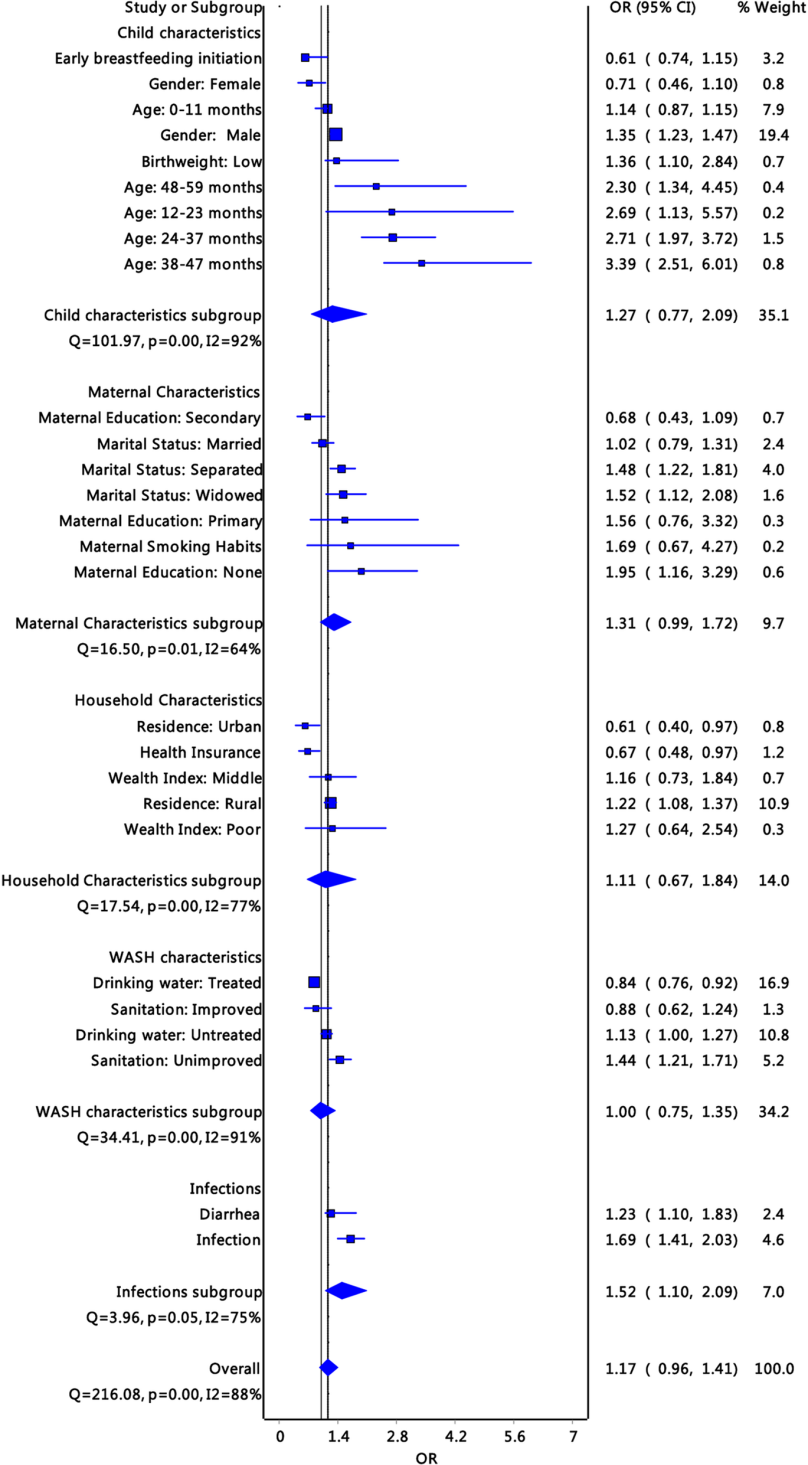
Subgroup analysis of determinants of stunting stratified as child, maternal, household, water, sanitation and hygiene characteristics, and infection

The results also indicate that socioeconomic inequality in stunting is intergenerational. The results showed that intergenerational transfers comprising maternal factors, maternal education, and low birth weight (*OR*: 1.36; *p* < 0.01; 95% *CI*: 1.1–2.84) were important determinants of stunting (Fig. 2). Several studies reported an association between marital status and stunting. The risk of stunting was high for children coming from households where parents were divorced (*OR*: 1.16; 95% *CI*: *p* = 0.004; 1.02–1.3), separated (*OR*: 1.48; *p* = 0.012; 95% *CI*: *p* = 0.002; 1.22–1.81), and widowed (OR: 1.52; *p* = 0.011; 95% *CI*: 1.12–2.08). Subgroup analysis by stratifying the determinants of child stunting into five characteristics showed that the likelihood of stunting due to child and maternal characteristics increases by 1.27 (*p* = 0.011; 95% *CI*: 0.77–2.09), and 1.31 (*p* < 0.001; 95% *CI*: 0.99–1.72) fold, respectively (Fig. 4).

When the model was reduced by removing nonsignificant factors, the likelihood of stunting due to child and maternal characteristics increased by 1.31 (*p* < 0.001; 95% *CI*: 0.60–2.85) and 1.41 (*p* < 0.01; 95% *CI*: 1.00–1.65) fold, respectively (Supplementary file 4). However, the observed heterogeneity (*I*^2^ > 90%) could not be reduced by subgroup analysis. A Luis Furuya–Kanamori (LFK) index of 1.45 suggests that there was minor asymmetry and a lack of publication bias (Supplementary file 5).

**Fig. 5:**
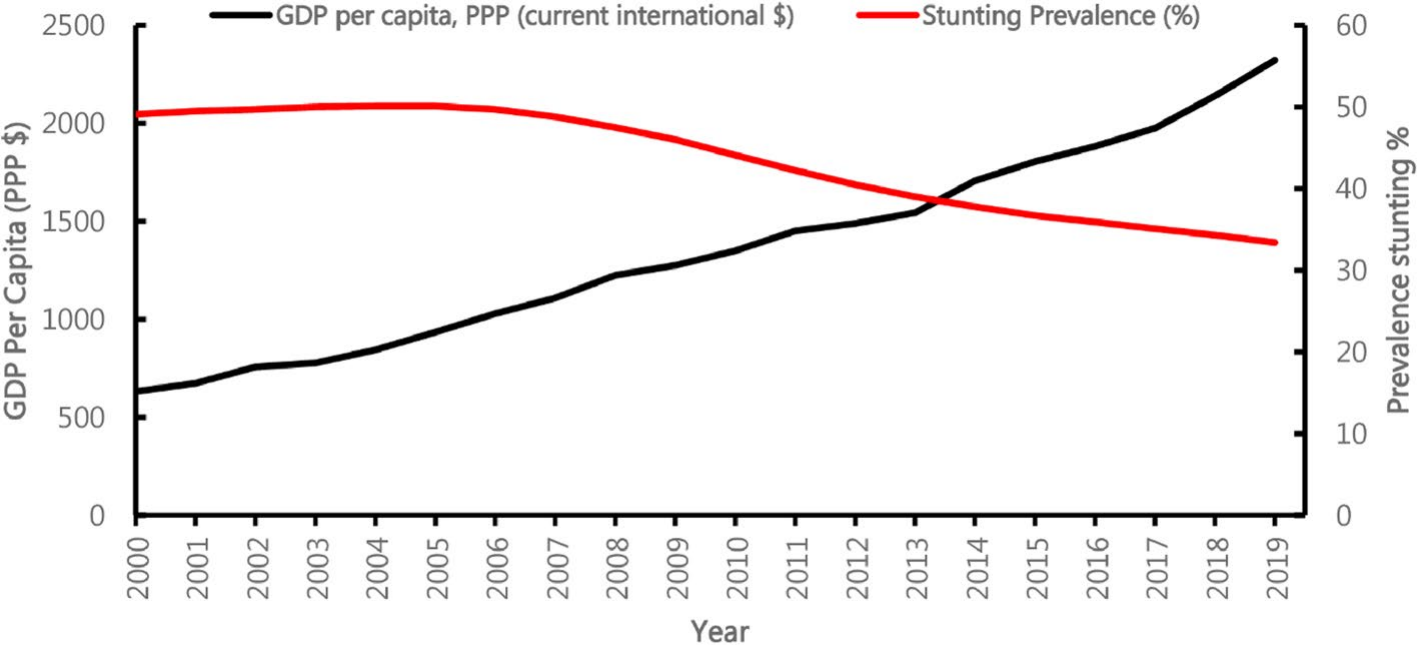
Changes in the GDP per capita and child stunting for the period 2000–2019

The results also indicate that there has been a reduction in the prevalence of stunting in Rwanda from 48.3% in 2000 to 33.1% in 2020. Furthermore, the reduction in the prevalence of child stunting during the study period coincided with an increase in gross domestic product (GDP) per capita in Rwanda (Fig. 5).

## Discussion

Understanding and examining the key drivers of stunting are essential for implementing tailored policies and programs for its reduction. Using the UNICEF conceptual framework, this study reviewed the available literature to evaluate the determinants of stunting among children under 5 years of age in Rwanda. Although the prevalence of stunting has been decreasing, the rate may have been slow, despite an increase in the country’s GDP. According to a study by Mary [39], a 10% increase in GDP per capita reduced the prevalence of child stunting by only 2.7%. Furthermore, Yaya et al. [40] suggested that for every US $1000 increase in GDP per capita, the odds of stunting decreased by 23%, an observation that does not apply to Rwanda. Our results further suggest the need to break the vicious circle of transgenerational poverty, improve economic status, enhance maternal health and education, and improve sanitation and access to treated water to reduce stunting among children under 5 years of age.

Our results show that unimproved sanitation and untreated drinking water are enablers of childhood stunting. Several studies conducted in low- and middle-income countries have suggested that children exposed to unsafe drinking water, poor sanitation, and poor hygiene are highly likely to have diarrheal diseases [41–44] and eventually infant morbidity and mortality [45]. In a study conducted by Torlesse et al. [46] in Indonesia, a significant association was observed between stunting and household sanitation. The outcome from our findings and findings from other studies when taken together indicates that implementation of improved nutrition strategies should, among other things, consider the improvement of sanitation facilities, as well as access to clean drinking water. The observed influence of unimproved sanitation on stunting underlines the importance of incorporating water, sanitation, and hygiene interventions into nutritional programs. Furthermore, this highlights the importance of addressing stunting programs using broader multisectoral approaches, unlike implementing nutrition-specific interventions.

Our results suggest that maternal factors such as education affect the likelihood of stunting among children. We have also observed from our study that children from households where mothers had no education or primary-level education were more likely to be stunted, an outcome that has also been reported in other studies [47, 48]. Improving the economic aspects of women, especially those from rural areas, is necessary to reduce the likelihood of stunting among these children. According to Galgamuwa et al. [49] and Kalinda et al. [47], higher education levels protect against stunting because educated women can make informed decisions about the nutritional value of food and understand the developmental and mental changes of a child. Corroborated by the study of Wirth et al. [50], Mensch et al. [51], and Waller et al. [52], higher maternal education influences key health outcomes and childcare practices. Thus, stunting reduction strategies would achieve desirable results if efforts to strengthen knowledge and skills are also incorporated, especially among young women and mothers from rural areas. This may play a critical role in reducing stunting by understanding ideal parenting patterns in infancy, childhood, and nutritional implementation.

Given that stunting was observed to be an intergenerational transfer in our study, addressing maternal-related factors is key to reducing childhood stunting. Several authors have observed that stunting is attributed to, among other factors, the mother’s education and health status as well as the accessibility and affordability of food [53, 54]. The inability to afford proper nutrition, especially during pregnancy, may lead to low birth weight [55, 56] and other irreversible and negative health outcomes among children, which also tend to impact their cognitive development, impaired academic performance, and prospects of future income [57–59]. The potential persistence of these problems in adulthood may lead to a circle of transgenerational poverty within communities [60]. Thus, there is a likelihood that maternal nutrition and health may be intimately linked to infant and child health and survival. Thus, there is a need for developing countries, such as Rwanda, to invest in improving maternal health to enhance stunting reduction.

According to Zerbo et al. [61], the coverage of water, sanitation, and hygiene (WASH) in SSA is low, increasing the risk of WASH-related diseases such as diarrhea. The results of our study indicate an association between diarrheal infection and stunting among children. Diarrhea among children leads to increased loss of essential nutrients and body fluids, reduced appetite, and intestinal absorption, which, in turn, leads to poor health and nutritional outcomes, as well as impaired growth [62]. The effect of diarrhea on stunting has also been confirmed in earlier studies by Odunayo and Oyewole [63] and Asfaw et al. [64], among others who conclude that the presence of diarrhea among children under-5 years old was associated with stunting. Our results and those of others suggest the need to improve environmental health to reduce the burden of diarrhea-causing pathogens. Progress in reducing diarrhea in Rwanda has been made through the introduction of three rounds of rotavirus vaccines administered to children 6, 10, and 14 weeks after delivery [65]. While these efforts are commendable, there is a need to enhance access to safe drinking water and to secure sanitation facilities.

This study showed that children from households with a poor wealth index (PWI) were more likely to be stunted than those from households with a rich wealth index (RWI). An earlier study by Mazoyer and Roudart [66] suggested that income and food prices influenced nutrition; thus, children from poor households remain at a higher risk of stunting when impacted by food prices and financial shocks. Poor households may have less income to provide high-quality nutritional food items and less access to basic health, thereby increasing the risk of infection. Our observation of the influence of HWI on the likelihood of stunting is consistent with studies conducted in Zambia [67, 68], Ethiopia [69], and elsewhere [70, 71]. Thus, improving childhood stunting requires addressing multiple factors, especially those that improve the socio-safety nets and financial structures of households, especially female-headed and widowed households, to help these potentially underprivileged households improve their food security.

Reducing the prevalence of stunting in SSA is a key developmental milestone. Rwanda aims to reduce it to 3% by 2050, as outlined in the Rwanda Vision 2050. Various factors must be comprehensively addressed to achieve this goal. Recently, Kalinda et al. [72] used a decomposition analysis to distinguish the potential determinants of stunting reduction in Rwanda. These authors suggested that child age, wealth index, maternal education, and the number of antenatal care visits were key to reducing stunting. However, the current study adds that achieving these also requires economic growth for communities. Similar results were found by Mary [39] who further suggested that increasing GDP per capita reduced the prevalence of child stunting by 2.7%. Elsewhere, Frongillo and Hanson [73], Smith and Haddad [74], and Haddad et al. [75] suggested that a higher per capita gross national product played an important role in improving stunting among children. However, with unequal distribution of income and wealth, poor and malnourished households may not be reached [76] leading to the persistence of the problem within these communities. This evidence suggests that stunting improvements are related to economic factors.

To achieve a significant reduction in child stunting within SSA, particularly in Rwanda and other countries where the prevalence remains higher than the regional average, policies that focus on addressing the immediate and underlying factors that influence stunting are needed. Several nutrition-based programs follow the path of locating children who are stunted and implement control measures. This traditional approach may miss the critical window of improving maternal health and nutrition at conception. Addressing stunting by focusing on infants may be costly and time-consuming and may, in the long run, affect the development of children and the economy of the country. There is a need to address stunting by improving the nutrition and health of mothers at conception until the child attains toddlerhood, especially in rural areas, where the likelihood of stunting among children is high compared to urban areas. In this way, children can grow rapidly and attain all developmental milestones while developing strong immunity against infections.

Although there is a paucity of data on the overall impact of individual economic outcomes and child stunting, several studies have demonstrated pathways associated with stunting and individual income [77, 78]. The results of our study show progress in reducing the prevalence of stunting in Rwanda. There is a need to sustain existing efforts, drive, and commitment to reduce child stunting further. According to Yaya et al. [40], improving women’s access to resources that enable them to engage in productive economic activities is an important step in reducing child stunting, as it creates growth opportunities. Child stunting affects economic growth and productivity through premature and preventable morbidity, mortality, and health expenditure [59, 79, 80], thus reducing human capital for production and productivity. Thus, investing in activities that improve household income among the poor is key to reducing stunting among children under the age of 5 years.

### Limitations and strengths of the study

One potential limitation may have been the unintentional exclusion of studies in the search that were written in language other than English, especially French. Rwanda initially used French as its official language before changing to English. Furthermore, the heterogeneous nature of reporting results from the included studies did not allow for statistical pooling of results, although this was addressed by reviewing the original DHS datasets that were used. In addition, given the substantial heterogeneity observed in this study, the observed determinants can be interpreted as potential factors that influence stunting and are not the only definitive factors. This is because the data that were extracted from the studies included in the meta-analysis focused on child, maternal, and household characteristics and applied logistic models. However, the multilevel nature of the DHS data suggests that the influence of community-level factors may not have been captured. However, this study considered some literature with countrywide data sources, indicating a relatively fair representation of the stunting situation and responses to address the causes at scale. Furthermore, the strength of this review is the general focus on understanding child stunting, considering the GDP of the country captured in this study.

## Conclusion

The results from the current study showed a diverse range of factors, varying degrees, and their association with stunting among children under 5 years of age in Rwanda. Reducing childhood stunting and maintaining the gains achieved between 2000 and 2020 depend on improving maternal health and nutrition before, during, and after pregnancy, enhancing social protection to improve the income of the poorest segments of the population, improving access to drinking water sources and sanitation, initiating early breastfeeding, and optimizing child complementary feeding. A multisectoral approach and implementation strategies that emphasize equity and target the most vulnerable population are needed.

Previous studies focusing on child stunting in Rwanda have mostly focused on conventional multidimensional maternal and child scarcity indicators, such as water, education, sanitation, shelter, information, and health in general. There is a need to understand the effects of sociocultural norms, beliefs, and practices, early maternal and child life exposures, maternal health, nutrition, and women’s access to resources on child stunting in Rwanda.

### Supplementary Information

**Additional file 1: Supplementary file 1.** Use of the UNICEF framework in the context of child stunting in Rwanda.

**Additional file 2: Supplementary file 2. **Quality assessment of the studies included in the meta-analysis.

**Additional file 3: Supplementary file 3. **Studies analysed in the meta-analysis based on the inclusion and exclusion criteria.

**Additional file 4: Supplementary file 4. **Forest plot of determinants of stunting after excluding non-significant factors.

**Additional file 5: Supplementary file 5. **Doi plot showing minor asymmetry and a lack of publication bias.

## Abbreviations

DHS: Demographic and Health Surveys
HWI: Household Wealth Index
UNICEF: United Nations International Children’s Emergency Fund
GDP: Gross domestic product
CI: Confidence interval
IVhet: Inverse variance heterogeneity model
FE: Fixed effect model
RE: Random effects model
LFK: Luis Furuya–Kanamori index
