# Supplementary material for: Leveraging multisectoral approach to understand the determinants of childhood stunting in Rwanda: a systematic review and meta-analysis
Source: Syst Rev. 2024 Jan 5;13:16. doi: 10.1186/s13643-023-02438-4 (PMC10768136; doi:10.1186/s13643-023-02438-4)
Supplement: Supplementary file 1 — Additional file 1: Supplementary file 1. Use of the UNICEF framework in the context of child stunting in Rwanda. [file 13643_2023_2438_MOESM1_ESM.docx]

The United Nations Children's Fund (UNICEF) Conceptual Framework was employed to pinpoint crucial indicators, and the Demographic and Health Surveys (DHS) 2000-2020 child recall datasets were scrutinized to recognize additional explanatory factors at the distal, intermediate, and proximal levels for child stunting in each of the reviewed papers that utilized DHS data. Additionally, the Exemplars analysis was deployed to facilitate the transformation of selected indicators as deemed appropriate for analysis.

The United Nations Children's Fund (UNICEF) has developed a comprehensive conceptual framework for understanding malnutrition, which is well-suited to the complex situation in Rwanda. This framework recognizes the multidimensional nature of malnutrition, incorporating factors beyond food intake, such as healthcare services, hygiene practices, and socioeconomic conditions. In common with many developing countries, Rwanda is confronted with a range of determinants of malnutrition. By adopting a holistic approach, it is possible to effectively disentangle the specific causal factors that are context specific to Rwanda. The socio-cultural context of Rwanda is of utmost importance in this analysis, and UNICEF's framework highlights the influence of social and cultural practices on nutritional outcomes. By adopting this approach, the study recognizes the significance of culture as a determinant of nutritional status, enabling a nuanced examination of the issue in Rwanda.

UNICEF's decision to employ a causal framework for investigating the determinants of stunted growth in Rwanda is a deliberate and considered strategy that considers the multifaceted nature of malnutrition, as well as the complex socio-cultural dynamics of the Rwandan environment.
