# Supplementary material for: Leveraging multisectoral approach to understand the determinants of childhood stunting in Rwanda: a systematic review and meta-analysis
Source: Syst Rev. 2024 Jan 5;13:16. doi: 10.1186/s13643-023-02438-4 (PMC10768136; doi:10.1186/s13643-023-02438-4)
Supplement: Supplementary file 2 — Additional file 2: Supplementary file 2. Quality assessment of the studies included in the meta-analysis. [file 13643_2023_2438_MOESM2_ESM.docx]

| **Author and year** | **Q1** | **Q2** | **Q3** | **Q4** | **Q5** | **Q6** | **Q7** | **Q8** | **Quality Index** |
| --- | --- | --- | --- | --- | --- | --- | --- | --- | --- |
| Ndagijimana et al. (2022) | 1 | 1 | 1 | 1 | 1 | 1 | 1 | 1 | 1 |
| Uwiringiyimana et al. (2022) | 1 | 1 | 1 | 1 | 1 | 1 | 1 | 1 | 1 |
| Rugema et al. (2022) | 1 | 1 | 1 | 1 | 1 | 1 | 1 | 1 | 1 |
| Umwali et al. (2022) | 1 | 1 | 0 | 0 | 1 | 1 | 1 | 1 | 0.75 |
| Nshimyiryo et al. (2019) | 1 | 1 | 1 | 1 | 1 | 1 | 1 | 1 | 1 |
| Binagwaho et al. (2020) | 1 | 1 | 1 | 1 | 1 | 1 | 1 | 1 | 1 |
| Agho et al. (2019) | 1 | 1 | 1 | 1 | 1 | 1 | 1 | 1 | 1 |
| Uwiringiyimana et al. (2019b) | 1 | 1 | 1 | 1 | 1 | 1 | 1 | 1 | 1 |
| Habimana and Biracyaza (2019) | 1 | 1 | 1 | 1 | 1 | 1 | 1 | 1 | 1 |
| Mutsindashyaka et al. (2020) | 1 | 1 | 1 | 1 | 1 | 1 | 1 | 1 | 1 |
| Sinharoy et al. (2016) | 1 | 1 | 1 | 1 | 1 | 1 | 1 | 1 | 1 |
| Binagwaho et al. (2014) | 1 | 1 | 1 | 1 | 1 | 1 | 1 | 1 | 1 |
| Kateera et al. (2015) | 1 | 1 | 1 | 1 | 1 | 1 | 1 | 1 | 1 |
| Rutayisire et al. (2020) | 1 | 1 | 1 | 1 | 0 | 1 | 1 | 1 | 0.875 |
| Bigirimana (2021) | 1 | 1 | 1 | 1 | 1 | 1 | 1 | 1 | 1 |
| Nsereko et al. (2018) | 1 | 1 | 1 | 1 | 1 | 1 | 1 | 1 | 1 |
| Weatherspoon et al. (2019) | 1 | 1 | 0 | 1 | 1 | 1 | 1 | 1 | 1 |
| Ngirabega et al. (2010) | 1 | 1 | 1 | 1 | 1 | 1 | 1 | 1 | 1 |
| Habyarimana et al. (2016) | 1 | 1 | 1 | 1 | 1 | 0 | 1 | 1 | 0.875 |
| Habyarimana (2016) | 1 | 1 | 1 | 1 | 1 | 1 | 1 | 1 |  |
| Uwiringiyimana et al. (2019a) | 1 | 1 | 1 | 1 | 1 | 1 | 1 | 1 | 11 |
| Lu et al. (2016) | 1 | 1 | 1 | 1 | 1 | 1 | 1 | 1 | 1 |
| Habimana et al. (2023) | 1 | 1 | 1 | 1 | 1 | 1 | 1 | 1 | 1 |
| Ndagijimana et al. (2023) | 1 | 1 | 1 | 1 | 1 | 0 | 1 | 1 | 0.875 |
| Niragire et al. (2022) | 1 | 1 | 1 | 1 | 1 | 0 | 1 | 1 | 0.875 |

**Assessment Questions**

|  | Yes | No | Unclear |
| --- | --- | --- | --- |
| 1. Were the criteria for inclusion in the sample clearly defined? | □ | □ | □ |
| 1. Were the study subjects and the setting described in detail? | □ | □ | □ |
| 1. Was the exposure measured in a valid and reliable way? | □ | □ | □ |
| 1. Were objective, standard criteria used for measurement of the condition? | □ | □ | □ |
| 1. Were confounding factors identified? | □ | □ | □ |
| 1. Were strategies to deal with confounding factors stated? | □ | □ | □ |
| 1. Were the outcomes measured in a valid and reliable way? | □ | □ | □ |
| 1. Was appropriate statistical analysis used? | □ | □ | □ |

**References**

Agho, K.E., Mukabutera, C., Mukazi, M., Ntambara, M., Mbugua, I., Dowling, M., et al. (2019). Moderate and severe household food insecurity predicts stunting and severe stunting among Rwanda children aged 6–59 months residing in Gicumbi district. *Maternal & child nutrition,* 15, e12767.

Bigirimana, J.B. (2021). Stunting among Under Five Years Old Children in Rwanda: Influences of Family Planning Status and Household Size and Composition. *Rwanda Journal of Medicine and Health Sciences,* 4, 112-130.

Binagwaho, A., Condo, J., Wagner, C., Ngabo, F., Karema, C., Kanters, S., et al. (2014). Impact of implementing performance-based financing on childhood malnutrition in Rwanda. *BMC public health,* 14, 1-8.

Binagwaho, A., Rukundo, A., Powers, S., Donahoe, K.B., Agbonyitor, M., Ngabo, F., et al. (2020). Trends in burden and risk factors associated with childhood stunting in Rwanda from 2000 to 2015: policy and program implications. *BMC public health,* 20, 83.

Habimana, J.d.D., Uwase, A., Korukire, N., Jewett, S., Umugwaneza, M., Rugema, L., et al. (2023). Prevalence and Correlates of Stunting among Children Aged 6–23 Months from Poor Households in Rwanda. *International journal of environmental research and public health,* 20, 4068.

Habimana, S., & Biracyaza, E. (2019). Risk factors of stunting among children under 5 years of age in the eastern and western provinces of Rwanda: analysis of Rwanda demographic and health survey 2014/2015. *Pediatric health, medicine and therapeutics,* 10, 115.

Habyarimana, F. (2016). Key determinants of malnutrition of children under five years of age in Rwanda: Simultaneous measurement of three anthropometric indices. *African Population Studies,* 30.

Habyarimana, F., Zewotir, T., Ramroop, S., & Ayele, D. (2016). Spatial Distribution of Determinants of Malnutrition of Children under Five Years in Rwanda: Simultaneous Measurement of Three Anthropometric Indices. *Journal of Human Ecology,* 54, 138-149.

Kateera, F., Ingabire, C.M., Hakizimana, E., Kalinda, P., Mens, P.F., Grobusch, M.P., et al. (2015). Malaria, anaemia and under-nutrition: three frequently co-existing conditions among preschool children in rural Rwanda. *Malaria Journal,* 14, 1-11.

Lu, C., Mejía-Guevara, I., Hill, K., Farmer, P., Subramanian, S., & Binagwaho, A. (2016). Community-based health financing and child stunting in rural Rwanda. *American journal of public health,* 106, 49-55.

Mutsindashyaka, T., Nshimyiryo, A., Beck, K., Kirk, C.M., Wilson, K., Mutaganzwa, C., et al. (2020). High Burden of Undernutrition among At-Risk Children in Neonatal Follow-Up Clinic in Rwanda. *Ann Glob Health,* 86, 125.

Ndagijimana, S., Kabano, I., & Ntaganda, J. (2022). Analysis of risk factors that influence stunting among Rwandan children under the age of five. *African Journal of Food, Agriculture, Nutrition and Development,* 22, 20480-20497.

Ndagijimana, S., Kabano, I.H., Masabo, E., & Ntaganda, J.M. (2023). Prediction of Stunting Among Under-5 Children in Rwanda Using Machine Learning Techniques. *J Prev Med Public Health,* 56, 41-49.

Ngirabega, J., Hakizimana, C., Wendy, L., Donnen, P., & Dramaix-Wilmet, M. (2010). Improving the management of a community based growth-monitoring program for children in rural Rwanda. *Revue D'epidemiologie et de Sante Publique,* 58, 111-119.

Niragire, F., Ndikumana, C., Nyirahabimana, M.G., & Mugemangango, C. (2022). Child stunting and associated risk factors in selected food-insecure areas in Rwanda: an analytical cross-sectional study. *Pan Afr Med J,* 43, 111.

Nsereko, E., Mukabutera, A., Iyakaremye, D., Umwungerimwiza, Y.D., Mbarushimana, V., & Nzayirambaho, M. (2018). Early feeding practices and stunting in Rwandan children: a cross-sectional study from the 2010 Rwanda demographic and health survey. *Pan Afr Med J,* 29, 157.

Nshimyiryo, A., Hedt-Gauthier, B., Mutaganzwa, C., Kirk, C.M., Beck, K., Ndayisaba, A., et al. (2019). Risk factors for stunting among children under five years: a cross-sectional population-based study in Rwanda using the 2015 Demographic and Health Survey. *BMC public health,* 19, 1-10.

Rugema, J., Mukantwari, J., Twagirayezu, I., Tuyisenge, M.J., Rutayisire, R., & Katende, G. (2022). Predictors and factors associated with stunting among under-five-year children: a cross-sectional population-based study in Rwanda of the 2014-2015 demographic and Health Survey. *African Health Sciences,* 4, 671-678.

Rutayisire, R., Kanazayire, C., Tuyisenge, G., & Munyanshongore, C. (2020). Trends in the Prevalence and Associated Contributing Factors of Stunting in Children Under Five Years of Age. Secondary Data Analysis of 2005, 2010 and 2014-2015 Rwanda Demographic and Health Surveys. *Rwanda Journal of Medicine and Health Sciences,* 3, 71-85.

Sinharoy, S.S., Schmidt, W.P., Cox, K., Clemence, Z., Mfura, L., Wendt, R., et al. (2016). Child diarrhoea and nutritional status in rural Rwanda: a cross‐sectional study to explore contributing environmental and demographic factors. *Tropical Medicine & International Health,* 21, 956-964.

Umwali, N., Kunyanga, C.N., & Kaindi, D.W.M. (2022). Determinants of stunting in children aged between 6–23 months in Musanze region, Rwanda. *Frontiers in Nutrition,* 9.

Uwiringiyimana, V., Ocké, M.C., Amer, S., & Veldkamp, A. (2019a). Predictors of stunting with particular focus on complementary feeding practices: A cross-sectional study in the northern province of Rwanda. *Nutrition,* 60, 11-18.

Uwiringiyimana, V., Osei, F., Amer, S., & Veldkamp, A. (2022). Bayesian geostatistical modelling of stunting in Rwanda: risk factors and spatially explicit residual stunting burden. *BMC public health,* 22, 1-14.

Uwiringiyimana, V., Veldkamp, A., & Amer, S. (2019b). Stunting spatial pattern in Rwanda: An examination of the demographic, socio-economic and environmental determinants. *Geospat Health,* 14.

Weatherspoon, D.D., Miller, S., Ngabitsinze, J.C., Weatherspoon, L.J., & Oehmke, J.F. (2019). Stunting, food security, markets and food policy in Rwanda. *BMC public health,* 19, 1-13.
