# Supplementary material for: Leveraging multisectoral approach to understand the determinants of childhood stunting in Rwanda: a systematic review and meta-analysis
Source: Syst Rev. 2024 Jan 5;13:16. doi: 10.1186/s13643-023-02438-4 (PMC10768136; doi:10.1186/s13643-023-02438-4)
Supplement: Supplementary file 3 — Additional file 3: Supplementary file 3. Studies analysed in the meta-analysis based on the inclusion and exclusion criteria. [file 13643_2023_2438_MOESM3_ESM.docx]

| **Author and year** | **Study objective** | **Data source** | **Study population** | **Results** |
| --- | --- | --- | --- | --- |
| Ndagijimana et al. (2022) | To assess factors of stunting, with the aim of revealing the system-wide impact of food insecurity on malnutrition. | DHS | Under 5 years children | Place of residence, altitude, wealth index and parental education level, maternal Body Mass Index (BMI), duration of breastfeeding, age and weight of the child at birth, sex, and birth order, occurrence of diarrhea, and parasitic infection were also associated with stunting. |
| Uwiringiyimana et al. (2022) | To study the spatial pattern of stunting in children less than five years considering anthropometric, socioeconomic, and demographic risk factors in Rwanda. In addition, we predict the spatial residuals effects to quantify the burden of stunting not accounted for by our geostatistical model. | DHS | Under 5 years children | Being male, lower birthweight (kg), non-exclusive breastfeeding, occurrence of diarrhea in the last two weeks, mothers with no or only primary education, living in a house with poor flooring material, reliance on a non-improved water source, and a low wealth index were the risk factors of stunting |
| Rugema et al. (2022) | To determine the predictors and factors associated with stunting among under-five children in Rwanda. | DHS | Under 5 years children | Child Age, sex, and place of residence, and household wealth index were associated with stunting. |
| Umwali et al. (2022) | Determining the implication of the infant and young child feeding practices in evaluating stunting in young children | Primary data | Under 5 years children | Minimum acceptable diet was found to be the stunting's predictor |
| Nshimyiryo et al. (2019) | Identifying risk factors of stunting in Rwanda | DHS | Under 5 years children | Boys, children ages 6–23 months, and children ages 24–59 months, compared to ages 0–6 months, low birth weight, low maternal height, primary education for mothers, illiterate mothers, history of not taking deworming medicine during pregnancy and poorest households were the determinants of stunting |
| Binagwaho et al. (2020) | Examine trends and potential risk factors associated with childhood stunting from 2000 to 2015 in Rwanda | DHS | Under 5 years children | Lowest wealth quintile, having a mother with limited education, having a mother that smoked, being male, and being of low-birth weight were associated with stunting. |
| Agho et al. (2019) | To examine the association between household food insecurity and stunting and severe stunting among Rwandan Children aged 6 to 59 months residing in Gicumbi Rwanda | Primary cross-sectional survey data | Under 5 years children | Moderate and severe household food insecurity were significantly higher among stunted children. Other predictors of stunting were gender (male children) and attendance of monthly growth monitoring sessions. |
| Uwiringiyimana et al. (2019b) | Study demographic, socio-economic and environmental factors determining the spatial pattern of stunting | DHS | Under 5 years children | Age of child, height of mother, maternal education, a child being male and birth weight and those were served by a rural market were associated with stunting. |
| Habimana and Biracyaza (2019) | determining the risk factors for stunting in children under 5 years of age in the Eastern and Western provinces of Rwanda | DHS | Under 5 years children | Antenatal care visits, sharing of toilets, gender of child, household wealth index, and breastfeeding were associated with stunting in eastern and western provinces. |
| Mutsindashyaka et al. (2020) | Assess the prevalence of undernutrition and associated factors among high-risk children born preterm, with low birth weight or other birth and neurodevelopmental injuries, who received nutritional support and clinical care follow-up in a Pediatric Development Clinic (PDC) in rural Rwanda | Primary cross-sectional survey | Under 5 years children | Small for gestational age, not having community health insurance (*mutuelle de santé*), being male, having a low weight at birth, increased age at enrolment in Pediatric Development Clinic, unknown gestational age or birth weight were the factors the increased the odds of stunting. |
| Sinharoy et al. (2016) | Explore associations of environmental and demographic factors with diarrhoea and nutritional status among children in Rusizi district, Rwanda. | Primary cross-sectional survey | Under 5 years children | Having an improved source of drinking water, treating drinking water using adequate methods,  having an improved sanitation facility and having a structurally complete sanitation facility were protective against stunting while practice open defecation was a risk factor of stunting. |
| Binagwaho et al. (2014) | assess the impact of performance-based financing (PBF) and other factors associated with the prevalence of three classifications of malnutrition (stunting, wasting and underweight) in children under-5 years in Rwanda. | Rwanda General Health and HIV household survey | Under 5 years children | Child age (12–23 months) was associated with stunting. Living in a district with performance-based financing was not found to be associated with stunting |
| Kateera et al. (2015) | Determining the prevalence of malaria parasitaemia, anaemia and under-nutrition among preschool age children in a rural Rwandan setting and evaluated for interactions between and risk determinants for these three conditions. | Primary cross-sectional survey | Under 5 years children | Stunting was associated with a history of infection (fever history) while factors that were protective against stunting were; living in a house where the Household head belonged to a higher economic group, and living in HHs that has a reported ownership of more than 1 Long lasting insecticide treated net. |
| Rutayisire et al. (2020) | To compare the prevalence rates of stunting in Rwanda using the Rwanda Demographic and Health Survey data of 2005, 2010 and 2014-2015 | DHS | Under 5 years children | The trends in the stunting prevalence rates among children under five years of age showed a decrease of 13% in stunting prevalence. Male children, children born with very small size, children born from mothers with no education and those born from poorest families more likely to be stunted. |
| Bigirimana (2021) | Examine the influences of household size and composition and family planning status on stunting among children under five in Rwanda | DHS | Under 5 years children | Number of children under five in the household, maternal age at childbirth, fertility preference, and unmet need for family planning were associated with stunting |
| Nsereko et al. (2018) | Evaluate the factors that contribute to childhood stunting by assessing feeding practices of Rwandans in children ≤ 2 years of age | DHS | Under 5 years children | High maternal educational level, high body mass index, and high household family income were protective against stunting while breastfeeding for at least 1 year, and solid food initiation increased the risks of stunting. |
| Weatherspoon et al. (2019) | Determine food intake patterns, dietary diversity, socio-economic risk and protective factors and quantify their influence on stunting in rural Rwandan children | Comprehensive Food Security and Vulnerability Analysis and Nutrition Survey | Under 5 years children | Male children were more likely to be stunted and children from rural areas had an increasing probability of being stunted. |
| Ngirabega et al. (2010) | To estimate the prevalence of malnutrition among children under five years of age and identify risk factors for malnutrition | Primary Nutritional survey | Under 5 years children | Absence of a mosquito net in the household, insufficient number of working adults in the household, the child being greater than 12 months, household managed by a man alone and being an orphan were the major risk factors of stunting |
| Habyarimana et al. (2016) | To identify the risk factors of malnutrition among children  under five years in Rwanda and produce the maps of prevalence of joint distribution of stunting, underweight and  wasting | DHS | Under 5 years children | Age of child, birth order, mother’s age at the birth, mother’s education level, gender of child,  birth weight, mother’s knowledge on nutrition, province, and wealth index were associated with stunting |
| Habyarimana (2016) | To identify the determinants of malnutrition of children under five years of age in Rwanda using height-for-age, weight-for-age and weight-for-height | DHS | Under 5 years children | Child’s age, birth order, maternal age at childbirth and education level, gender of the child, birth weight, province, mother’s knowledge of nutrition and wealth index were risk factors stunting |
| Uwiringiyimana et al. (2019a) | To review the factors associated with stunting in the northern province of Rwanda by assessing anthropometric status, dietary intake, and overall complementary feeding practices | Primary cross-sectional study | Children aged 5-30 months | Child age, exclusive breastfeeding, use of deworming tablets, caregiver BMI, and dietary zinc intake were predictors of stunting. |
| Lu et al. (2016) | To assess the likelihood of rural children (aged 6–24 months) being stunted according to whether they were enrolled in *Mutuelles*, a community-based health-financing program. | District Health System Strengthening Tool (DHSST) | Children aged 6–24 months | Enrolment into Mutuelles was protective against stunting |
| Habimana et al. (2023) | To evaluate the prevalence and associated factors of stunting among children aged 6–23 months from poor households in Rwanda | Primary cross-sectional study | Children aged 6–23 months | Children aged 19–23 and 13–18 months were at high risk of stunting. Children whose mothers were not exposed to physical violence, those whose fathers were working, those whose parents were both working, and children whose mothers demonstrated good hand washing practice were less likely to be stunted. |
| Ndagijimana et al. (2023) | To develop a model for predicting stunting in Rwandan children | DHS | Under 5 years children | mother’s height, watching television, the baby’s age, province, size at childbirth, and mother’s education were the most important predictors of stunting status |
| Niragire et al. (2022) | To ascertain the key factors that protect children from stunting in these poorest areas, where stunting rates are lower than expected. | DHS | Under 5 years children | Birth weight of at least 2.5 kg, a high household economic status, urban residence and health insurance coverage protective against child stunting while children aged one year and above and female-headed households were risk factors of stunting. |

**References**

Agho, K.E., Mukabutera, C., Mukazi, M., Ntambara, M., Mbugua, I., Dowling, M., et al. (2019). Moderate and severe household food insecurity predicts stunting and severe stunting among Rwanda children aged 6–59 months residing in Gicumbi district. *Maternal & child nutrition,* 15, e12767.

Bigirimana, J.B. (2021). Stunting among Under Five Years Old Children in Rwanda: Influences of Family Planning Status and Household Size and Composition. *Rwanda Journal of Medicine and Health Sciences,* 4, 112-130.

Binagwaho, A., Condo, J., Wagner, C., Ngabo, F., Karema, C., Kanters, S., et al. (2014). Impact of implementing performance-based financing on childhood malnutrition in Rwanda. *BMC public health,* 14, 1-8.

Binagwaho, A., Rukundo, A., Powers, S., Donahoe, K.B., Agbonyitor, M., Ngabo, F., et al. (2020). Trends in burden and risk factors associated with childhood stunting in Rwanda from 2000 to 2015: policy and program implications. *BMC public health,* 20, 83.

Habimana, J.d.D., Uwase, A., Korukire, N., Jewett, S., Umugwaneza, M., Rugema, L., et al. (2023). Prevalence and Correlates of Stunting among Children Aged 6–23 Months from Poor Households in Rwanda. *International journal of environmental research and public health,* 20, 4068.

Habimana, S., & Biracyaza, E. (2019). Risk factors of stunting among children under 5 years of age in the eastern and western provinces of Rwanda: analysis of Rwanda demographic and health survey 2014/2015. *Pediatric health, medicine and therapeutics,* 10, 115.

Habyarimana, F. (2016). Key determinants of malnutrition of children under five years of age in Rwanda: Simultaneous measurement of three anthropometric indices. *African Population Studies,* 30.

Habyarimana, F., Zewotir, T., Ramroop, S., & Ayele, D. (2016). Spatial Distribution of Determinants of Malnutrition of Children under Five Years in Rwanda: Simultaneous Measurement of Three Anthropometric Indices. *Journal of Human Ecology,* 54, 138-149.

Kateera, F., Ingabire, C.M., Hakizimana, E., Kalinda, P., Mens, P.F., Grobusch, M.P., et al. (2015). Malaria, anaemia and under-nutrition: three frequently co-existing conditions among preschool children in rural Rwanda. *Malaria Journal,* 14, 1-11.

Lu, C., Mejía-Guevara, I., Hill, K., Farmer, P., Subramanian, S., & Binagwaho, A. (2016). Community-based health financing and child stunting in rural Rwanda. *American journal of public health,* 106, 49-55.

Mutsindashyaka, T., Nshimyiryo, A., Beck, K., Kirk, C.M., Wilson, K., Mutaganzwa, C., et al. (2020). High Burden of Undernutrition among At-Risk Children in Neonatal Follow-Up Clinic in Rwanda. *Ann Glob Health,* 86, 125.

Ndagijimana, S., Kabano, I., & Ntaganda, J. (2022). Analysis of risk factors that influence stunting among Rwandan children under the age of five. *African Journal of Food, Agriculture, Nutrition and Development,* 22, 20480-20497.

Ndagijimana, S., Kabano, I.H., Masabo, E., & Ntaganda, J.M. (2023). Prediction of Stunting Among Under-5 Children in Rwanda Using Machine Learning Techniques. *J Prev Med Public Health,* 56, 41-49.

Ngirabega, J., Hakizimana, C., Wendy, L., Donnen, P., & Dramaix-Wilmet, M. (2010). Improving the management of a community based growth-monitoring program for children in rural Rwanda. *Revue D'epidemiologie et de Sante Publique,* 58, 111-119.

Niragire, F., Ndikumana, C., Nyirahabimana, M.G., & Mugemangango, C. (2022). Child stunting and associated risk factors in selected food-insecure areas in Rwanda: an analytical cross-sectional study. *Pan Afr Med J,* 43, 111.

Nsereko, E., Mukabutera, A., Iyakaremye, D., Umwungerimwiza, Y.D., Mbarushimana, V., & Nzayirambaho, M. (2018). Early feeding practices and stunting in Rwandan children: a cross-sectional study from the 2010 Rwanda demographic and health survey. *Pan Afr Med J,* 29, 157.

Nshimyiryo, A., Hedt-Gauthier, B., Mutaganzwa, C., Kirk, C.M., Beck, K., Ndayisaba, A., et al. (2019). Risk factors for stunting among children under five years: a cross-sectional population-based study in Rwanda using the 2015 Demographic and Health Survey. *BMC public health,* 19, 1-10.

Rugema, J., Mukantwari, J., Twagirayezu, I., Tuyisenge, M.J., Rutayisire, R., & Katende, G. (2022). Predictors and factors associated with stunting among under-five-year children: a cross-sectional population-based study in Rwanda of the 2014-2015 demographic and Health Survey. *African Health Sciences,* 4, 671-678.

Rutayisire, R., Kanazayire, C., Tuyisenge, G., & Munyanshongore, C. (2020). Trends in the Prevalence and Associated Contributing Factors of Stunting in Children Under Five Years of Age. Secondary Data Analysis of 2005, 2010 and 2014-2015 Rwanda Demographic and Health Surveys. *Rwanda Journal of Medicine and Health Sciences,* 3, 71-85.

Sinharoy, S.S., Schmidt, W.P., Cox, K., Clemence, Z., Mfura, L., Wendt, R., et al. (2016). Child diarrhoea and nutritional status in rural Rwanda: a cross‐sectional study to explore contributing environmental and demographic factors. *Tropical Medicine & International Health,* 21, 956-964.

Umwali, N., Kunyanga, C.N., & Kaindi, D.W.M. (2022). Determinants of stunting in children aged between 6–23 months in Musanze region, Rwanda. *Frontiers in Nutrition,* 9.

Uwiringiyimana, V., Ocké, M.C., Amer, S., & Veldkamp, A. (2019a). Predictors of stunting with particular focus on complementary feeding practices: A cross-sectional study in the northern province of Rwanda. *Nutrition,* 60, 11-18.

Uwiringiyimana, V., Osei, F., Amer, S., & Veldkamp, A. (2022). Bayesian geostatistical modelling of stunting in Rwanda: risk factors and spatially explicit residual stunting burden. *BMC public health,* 22, 1-14.

Uwiringiyimana, V., Veldkamp, A., & Amer, S. (2019b). Stunting spatial pattern in Rwanda: An examination of the demographic, socio-economic and environmental determinants. *Geospat Health,* 14.

Weatherspoon, D.D., Miller, S., Ngabitsinze, J.C., Weatherspoon, L.J., & Oehmke, J.F. (2019). Stunting, food security, markets and food policy in Rwanda. *BMC public health,* 19, 1-13.
