# Supplementary figures and images for: Leveraging multisectoral approach to understand the determinants of childhood stunting in Rwanda: a systematic review and meta-analysis
Source: Syst Rev. 2024 Jan 5;13:16. doi: 10.1186/s13643-023-02438-4 (PMC10768136; doi:10.1186/s13643-023-02438-4)

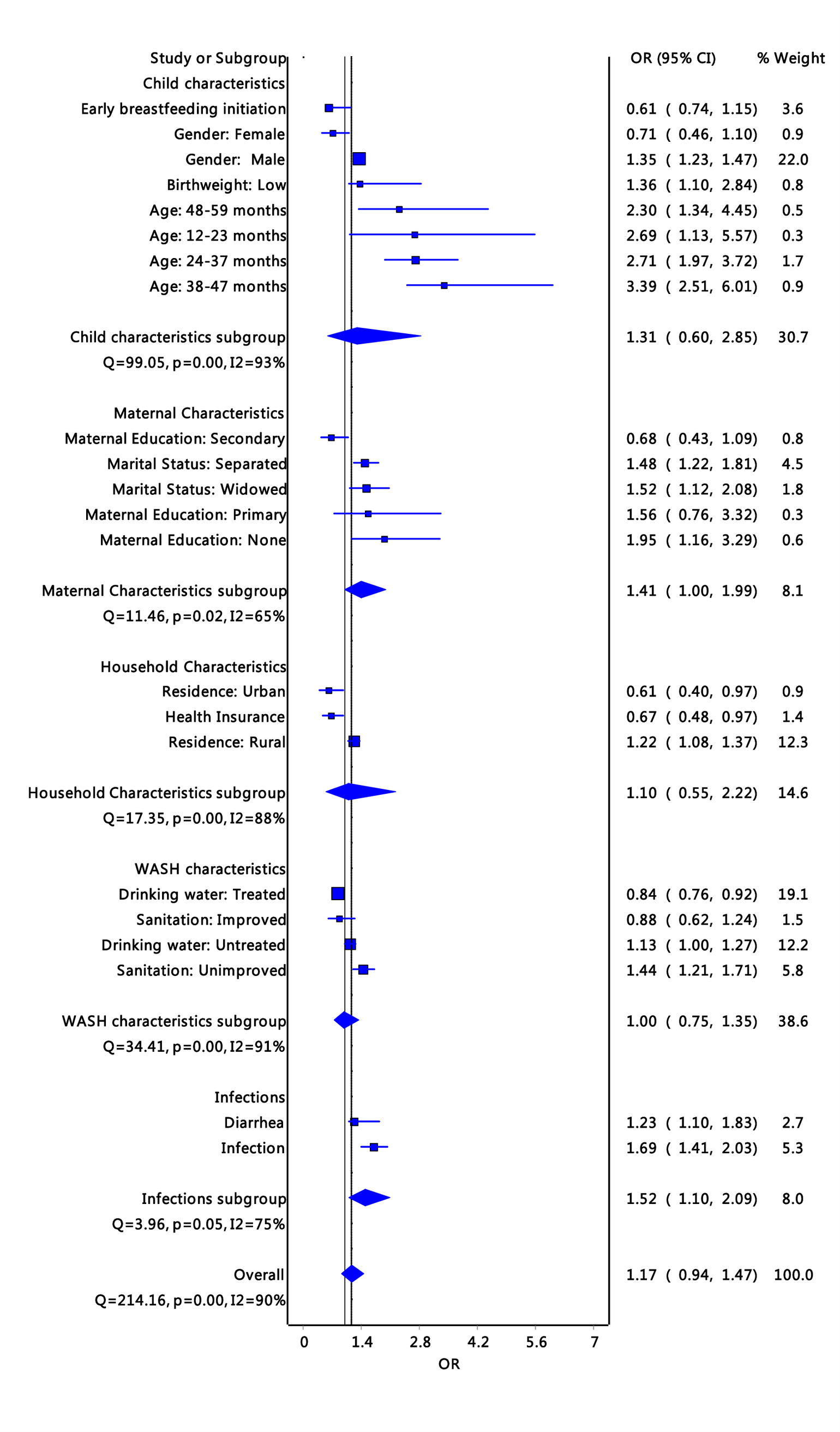

Supplement: Supplementary file 4 — Additional file 4: Supplementary file 4. Forest plot of determinants of stunting after excluding non-significant factors. [file 13643_2023_2438_MOESM4_ESM.tif]

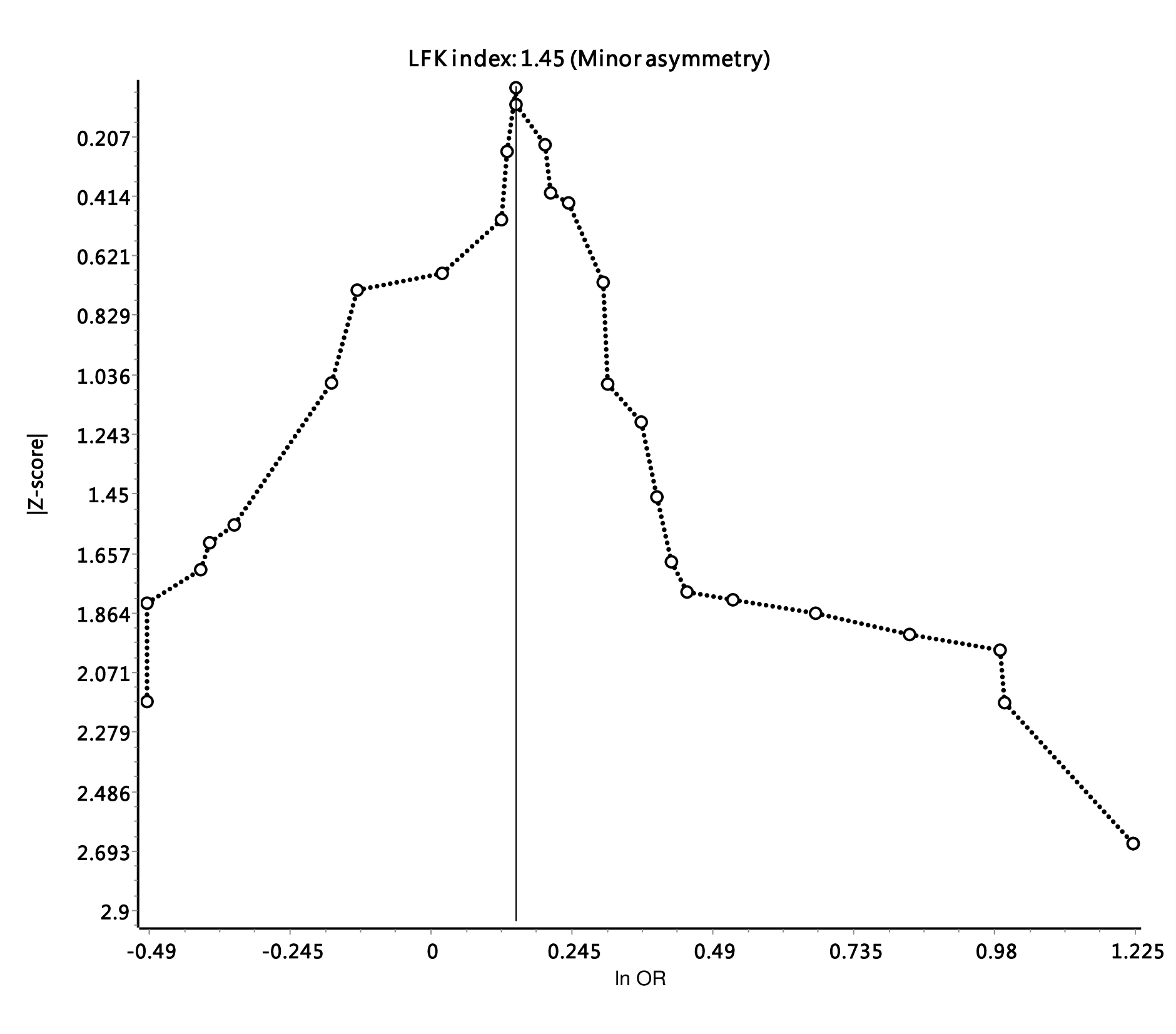

Supplement: Supplementary file 5 — Additional file 5: Supplementary file 5. Doi plot showing minor asymmetry and a lack of publication bias. [file 13643_2023_2438_MOESM5_ESM.tif]
